# Supplementary material for: The Construction and Exploration of a Comprehensive MicroRNA Centered Regulatory Network in Foxtail Millet (Setaria italica L.)
Source: Front Plant Sci. 2022 May 6;13:848474. doi: 10.3389/fpls.2022.848474 (PMC9121102; doi:10.3389/fpls.2022.848474)
Supplement: Supplementary file 2 [file Data_Sheet_2.zip › Sit-miR398b_Seita.9G341500.1_222_TPlot.pdf]

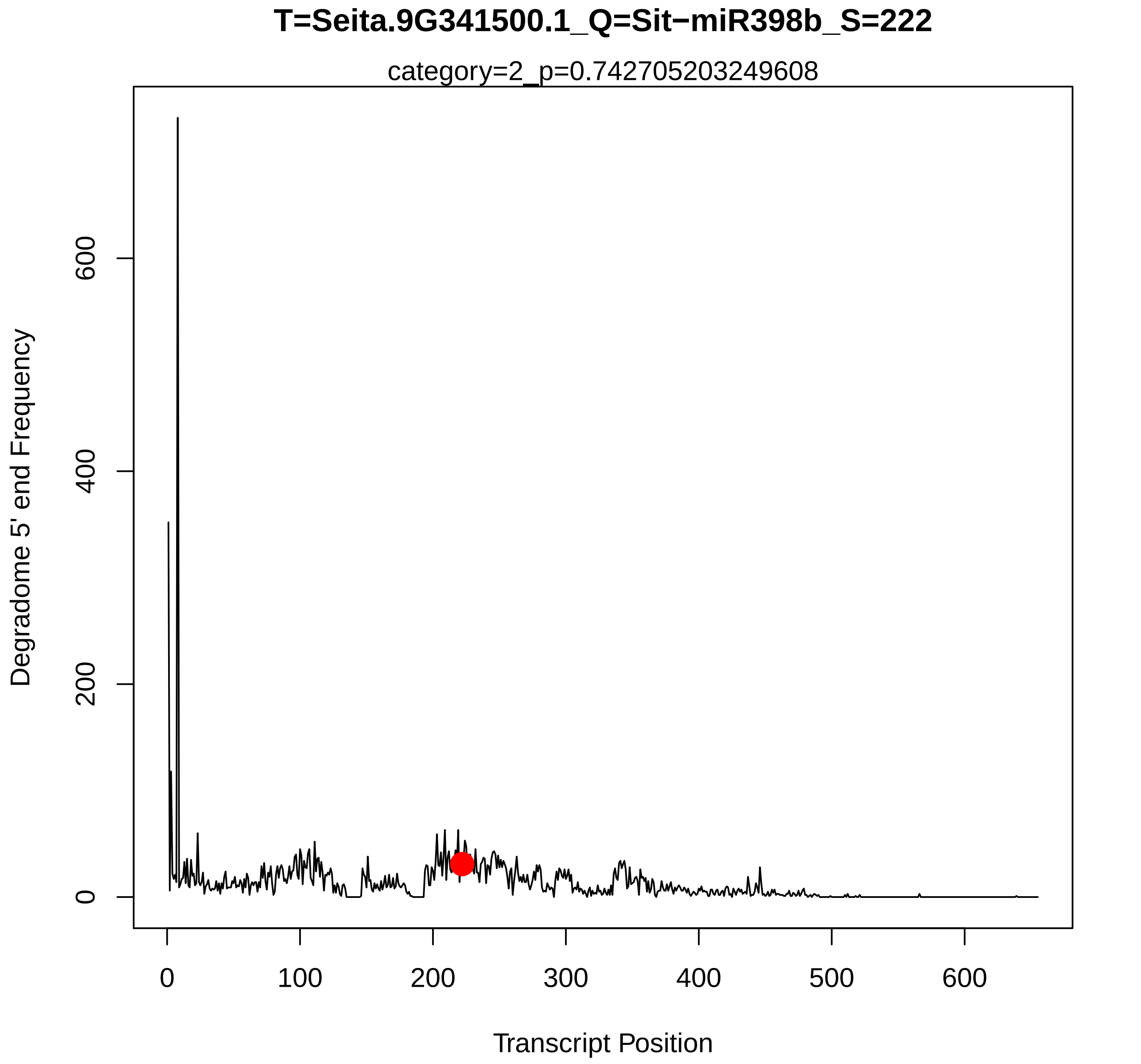
T=Seita.9G341500.1_Q=Sit−miR398b_S=222
category=2_p=0.742705203249608
Degradome 5' end Frequency
0
200
400
600
0
100
200
300
400
500
600
Transcript Position
